# A case report of bilateral persistent sciatic artery with thrombosis in a 47-year-old woman: A rare vascular anomaly

**DOI:** 10.1016/j.ijscr.2024.110791

**Published:** 2024-12-26

**Authors:** Ahmad Hoseinzadeh, Hamid Zaferani Arani, Ali Tajaddini, Shekoofeh Rahimi, Fatemeh Mirparsa, Sedighe Hooshmandi

**Affiliations:** a Department of Surgery, Shiraz University of Medical Sciences, Shiraz, Iran; b Department of Health Policy, Faculty of Health, Scientific Pole of Health Sciences Education, Tehran Medical Sciences, Tehran University of Medical Sciences, Tehran, Iran; c Medical Imaging Research Center, Department of Radiology, Shiraz University of Medical Sciences, Shiraz, Iran

**Keywords:** Persistent sciatic artery (PSA), Vascular anomaly, Thrombosis, Case report

## Abstract

**Introduction:**

The persistent sciatic artery (PSA) is a rare congenital vascular anomaly that arises when the embryonic axial artery fails to regress, potentially leading to serious complications such as limb ischemia.

**Case presentation:**

We report the case of a 47-year-old woman with a history of essential hypertension and recent hormonal treatment for uterine fibroids. She developed acute limb ischemia due to bilateral PSA thrombosis, which was confirmed through comprehensive imaging. A multidisciplinary team implemented a management strategy that included anticoagulation therapy and close monitoring, resulting in significant improvement in her symptoms.

**Clinical discussion:**

The incidence of PSA thrombosis is low, estimated at around 0.04 % to 0.06 %, with bilateral involvement in approximately 30 % of cases. This case underscores the importance of individualized treatment plans and highlights the need for increased awareness and research into PSA to optimize clinical outcomes, particularly in resource-limited settings.

**Conclusion:**

This report emphasizes the critical role of early diagnosis and a tailored approach to management in patients with PSA.

## Highlights

• A persistent sciatic artery (PSA) is an uncommon congenital vascular anomaly.
• This condition results in the PSA remaining as the main arterial supply.
• PSA is susceptible to early atherosclerosis and distal embolization.
• Computed tomography angiography (CTA) is effective in detecting PSA.
• We present a rare case study detailing PSA thrombosis and its management by anticoagulant therapy.

## 1 Introduction

The persistent sciatic artery (PSA) is a rare congenital vascular anomaly characterized by the failure of the embryonic axial artery to regress during fetal development [1]. Normally, the sciatic artery serves as the primary blood supply to the lower limb, transitioning to the more common femoral artery by the 12th week of gestation [2]. However, in cases of PSA, this transformation does not occur, resulting in the persistence of the sciatic artery as the main arterial conduit. The incidence of PSA is exceptionally low, estimated at approximately 0.04 % to 0.06 %, and it does not show significant gender predisposition [3]. Notably, about 30 % of cases exhibit bilateral involvement, complicating clinical management. The embryological development of PSA is linked to disruptions in the normal regression of the sciatic artery, which can lead to various anatomical variations, including the presence of accessory vessels that complicate the vascular supply to the lower limb [4]. Clinically, PSA is significant due to its predisposition to complications such as early atherosclerosis, distal embolization, and aneurysmal degeneration [5]. Patients may present with diverse symptoms, including limb ischemia due to thromboembolic events or neuralgia from sciatic nerve compression, which can result in functional impairment. Computed tomography angiography (CTA) has emerged as an effective diagnostic tool for identifying PSAs, even in cases where the artery is fully occluded, and it assists in evaluating associated complications [5]. Management options for symptomatic PSAs typically include open surgical intervention or endovascular treatment, tailored to the specific symptoms and anatomical considerations of the patient [6]. However, literature on the management of PSA is limited, with most recommendations derived from case reports and small series. In this report, we present a case study of a woman with bilateral PSA thrombosis, exploring the intricacies of her vascular anomaly and the subsequent management through anticoagulant therapy while discussing existing literature and the need for further research.

## 2 Case presentation

**Fig. 1:**
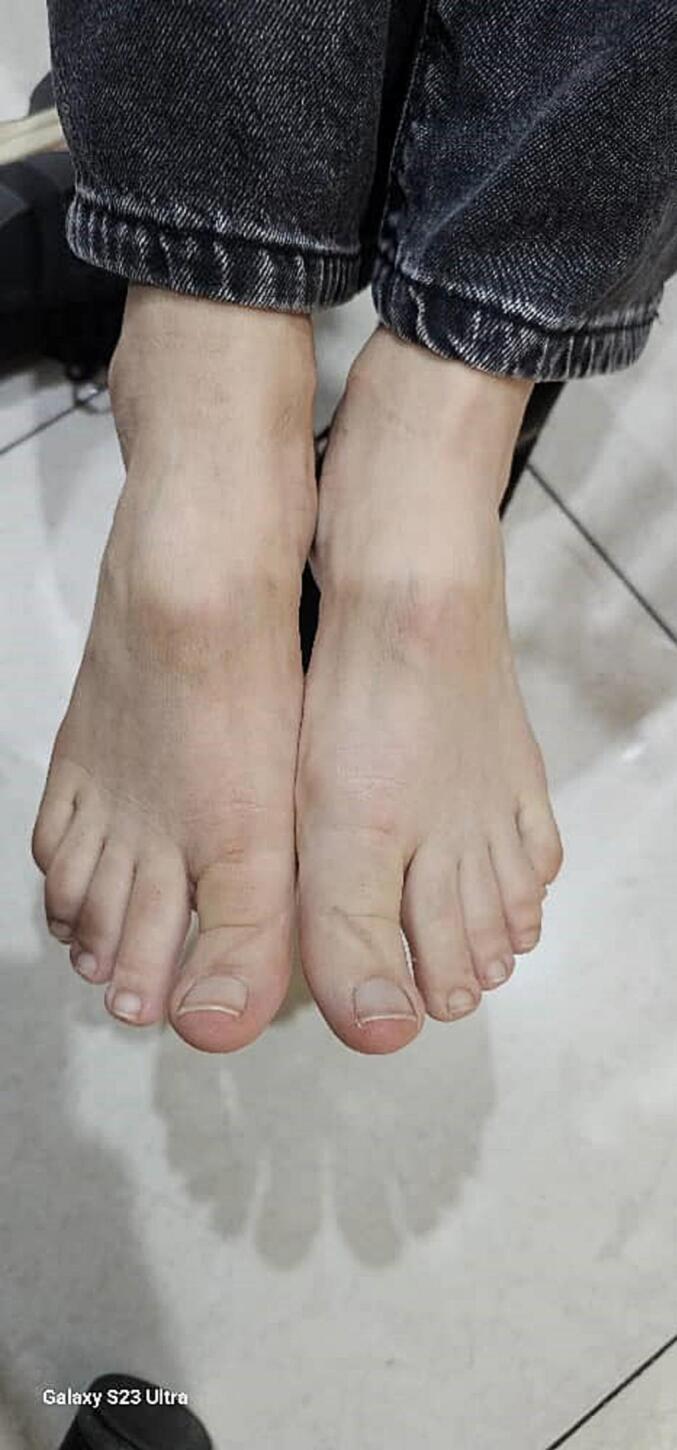
Clinical photograph showing left leg discoloration in the forefoot area, and mottling in the toes of her left foot.

**Fig. 2:**
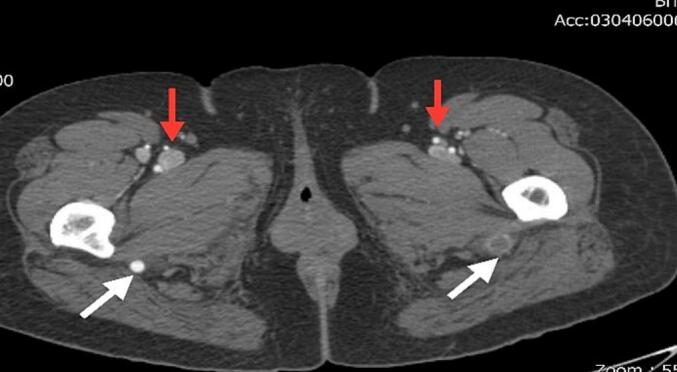
Axial CT angiogram demonstrated bilateral persistent sciatic arteries (white arrows) with aneurysmal dilatation left sciatic artery and complete thrombosis proximal part, femoral arteries (red arrows) are hypoplastic.

**Fig. 3:**
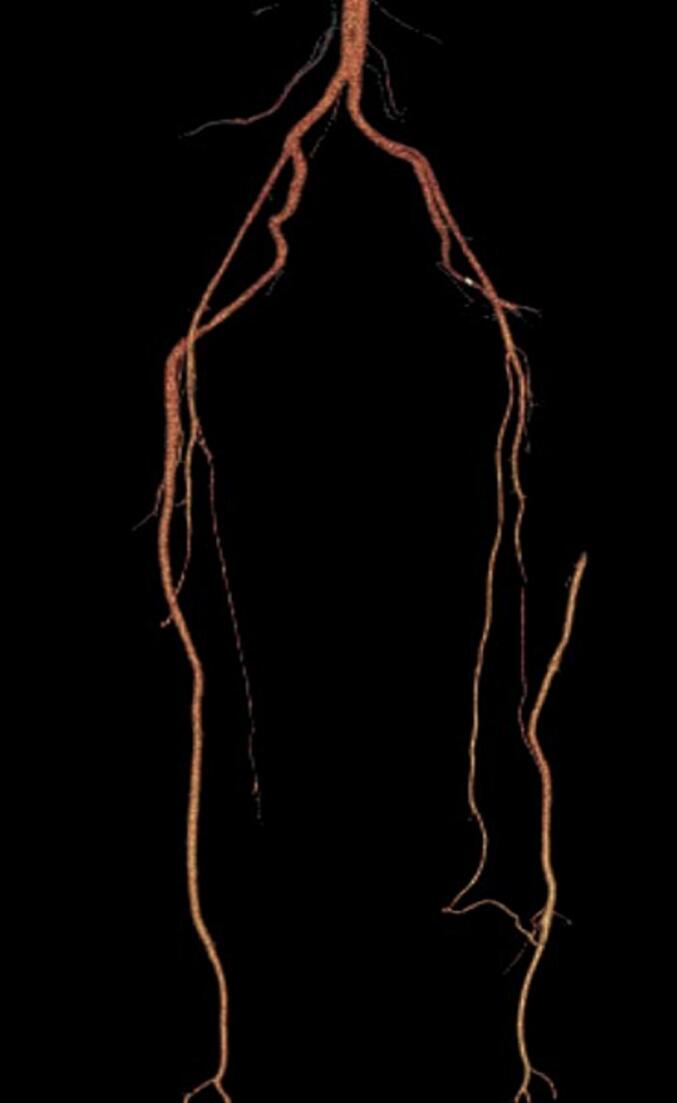
Coronal view 3D CT reconstruction of the arteries from hip to thigh, demonstrated hypertrophy of right sciatic artery and thrombosis proximal of left sciatic artery, mid part filled with collateral vessels.

**Fig. 4:**
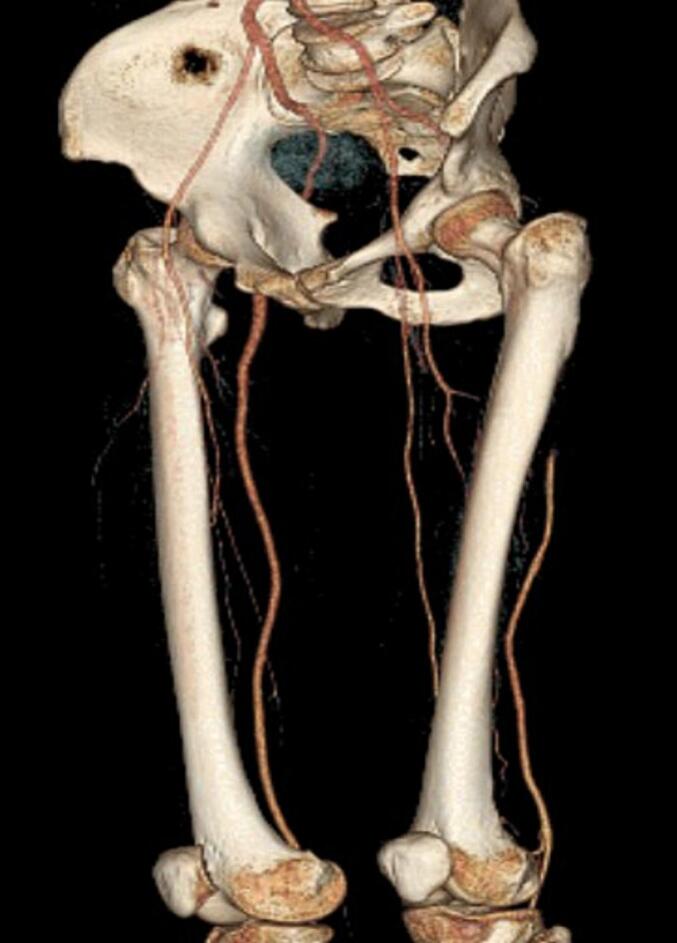
Anterior view of 3D CT reconstruction arteries.

**Fig. 5:**
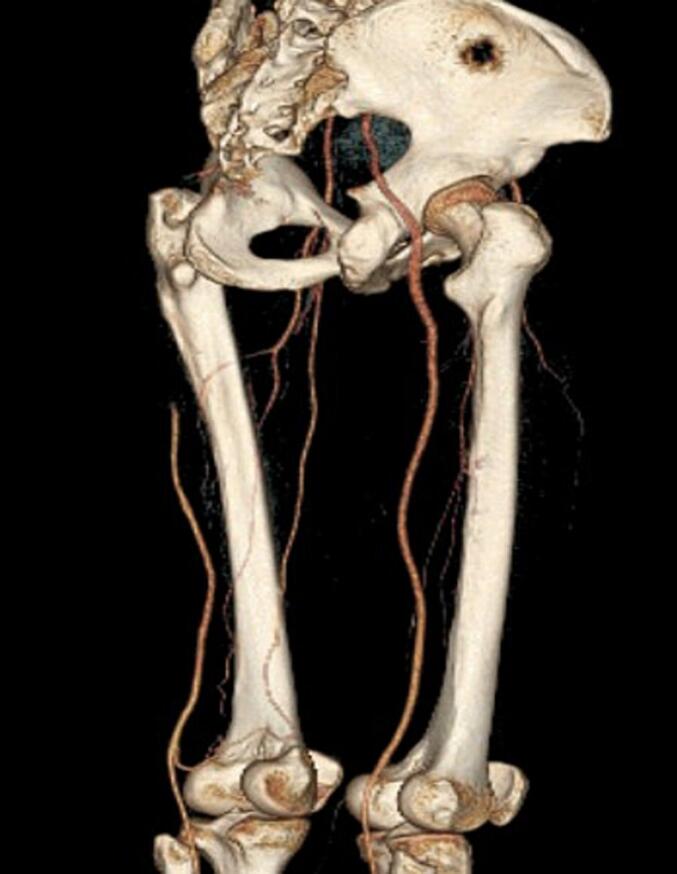
Posterior lateral view 3D CT reconstruction.

We report the case of a 47-year-old female patient with a significant medical history of essential hypertension, managed with valsartan. Approximately two weeks before her presentation with vascular symptoms, she had been treated with a Diphereline (leuprolide acetate) injection for uterine fibroids measuring 2–3 cm. The patient was not on any other medications that would predispose her to thrombosis and had no prior history of thromboembolic events. Two weeks post-injection, the patient developed acute left leg pain accompanied by discoloration in the forefoot, specifically mottling of the toes. The pain, initially mild, progressed to severe rest pain in the calf and distal foot, prompting her to seek medical attention. Upon examination, there was a notable absence of a pulse in the left lower extremity, and the mottled appearance indicated potential ischemia (Fig. 1). Differential diagnoses considered included acute limb ischemia secondary to thrombosis or embolism and potential entrapment or compression syndromes related to the recent injection. To further evaluate her condition, CT angiography was performed, revealing monophasic flow changes in the proximal left leg, indicative of thrombosis. The imaging demonstrated the presence of bilateral PSA and confirmed thrombosis in the proximal left leg (Fig. 2, Fig. 3, Fig. 4, Fig. 5). Other potential causes of her symptoms, such as vascular occlusions from embolic sources, were excluded. A formal diagnosis of bilateral PSA thrombosis was established.

Given the patient's overall state and the severity of her ischemic symptoms, anticoagulant therapy was initiated as the primary method of management. The decision against surgical intervention was significantly influenced by the resource-limited setting, where the availability of surgical expertise and facilities may be constrained. Additionally, the patient's clinical stability suggested that non-operative management could be effective, especially with the timely initiation of anticoagulants.

On her follow-up visit, the patient reported significant improvement, with a marked reduction in both mottling and rest pain. By the one-month follow-up, her claudication distance improved to 50 m, indicating a satisfactory recovery. The patient was continued on a non-operative management plan, and she was advised to avoid hormonal medications that could exacerbate her condition. She was prescribed atorvastatin for dyslipidemia, valsartan (Valsomix) for blood pressure control, and apixaban for anticoagulation to prevent future thrombotic events.

## 3 Discussion

The management of PSA is challenging due to its rarity and the limited evidence available in the literature. Most existing studies focus on case reports and small case series, which highlight various management strategies but lack the robust data needed to establish standardized treatment protocols [7,8]. For instance, while some authors advocate for surgical intervention in symptomatic cases, others suggest conservative management with anticoagulation as a viable option, particularly in resource-limited settings [[8], [9], [10]].

A review of the literature reveals that the primary treatment approach for symptomatic PSA often includes anticoagulation therapy, particularly in cases where thromboembolism has occurred [3,11]. This conservative management strategy was chosen for our patient due to her clinical stability and the resource-limited setting where surgical options might not be readily available [12]. However, the lack of large-scale studies means that clinicians must rely on anecdotal evidence when deciding on treatment plans.

**Fig. 6:**
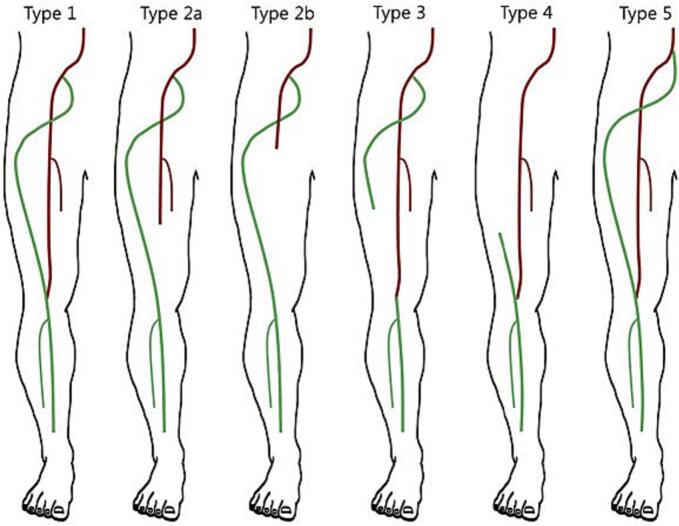
PSAs were classified into five types according to the Pillet classification, modified by Gauffre [13].

The Pillet classification, modified by Gauffre (Fig. 6), delineates PSA into distinct types, which play a crucial role in guiding management decisions and understanding the anatomical variations associated with the anomaly [13]. These classifications help clinicians assess the potential risks and treatment strategies based on specific anatomical presentations. For example, type 2 patients, with an incompletely developed femoral artery, may be at greater risk for thrombotic complications due to inadequate collateral circulation. Recognizing these classifications aids in prognostication and tailoring individualized treatment plans that address the unique vascular architecture of each patient [14].

Given the limited evidence, there is a pressing need for further research to establish comprehensive management guidelines for PSA. Future studies should focus on multicenter registries to collect data on patient outcomes, treatment responses, and long-term follow-up. This could help identify best practices and standardize care for patients with this condition. Additionally, the development of clinical trials evaluating the efficacy of various treatment modalities, including surgical versus conservative management, would provide valuable insights into optimizing patient outcomes.

The work has been reported in line with the SCARE criteria [15].

## 4 Conclusion

PSA is an uncommon vascular anomaly that poses significant clinical challenges and requires vigilant monitoring and management due to its potential for life-threatening complications. This case highlights the critical importance of early diagnosis and a multidisciplinary approach to managing PSA. Clinicians must be aware of the implications of this anomaly, as it can lead to serious health issues or serve as a vital alternative blood supply for the limb. The clinical lessons learned from this case include recognizing the signs and symptoms associated with PSA and the importance of considering conservative management options, such as anticoagulant therapy, particularly in patients with recent hormonal treatments. To address the gaps in the literature, future research should focus on developing comprehensive management guidelines for PSA, especially in resource-limited settings, to standardize care and improve patient outcomes. Additionally, further studies are warranted to explore the long-term outcomes of conservative versus surgical management in patients with PSA, as well as the potential impact of various treatment modalities on quality of life and functional status.

## Patient consent

Written informed consent was obtained from the patient for publication and any accompanying images. A copy of the written consent is available for review by the Editor-in-Chief of this journal on request.

## CRediT authorship contribution statement

Ahmad Hoseinzadeh: Conceptualization, methodology, software, writing, editing.
Hamid Zaferani Arani: Data curation, writing-original draft preparation.
Ali Tajaddini, Shekoofeh Rahimi, Fatemeh Mirparsa: Data curation, writing-original draft preparation.
Sedighe Hooshmandi: Patient management, supervision, writing, editing.

## Ethical approval

The patient provided written consent for this case report, which is presented in an anonymized format. According to the guidelines at our school (School of Medicine, Shiraz University of Medical Sciences, Shiraz, Iran), single case reports do not require separate ethical approval, as they do not contain identifying information and do not involve experimental treatment.

## Guarantor

Dr. Sedighe Hooshmandi
Dr. Hamid Zaferani Arani.

## Funding

Not applicable.

## Declaration of competing interest

The authors declare that they have no competing interests.

## Data Availability

The clinical documentation of the presented case cannot be made public due to the detailed identifiable information of the patient.
